# Transcriptome Analysis of Small Molecule–Mediated Astrocyte-to-Neuron Reprogramming

**DOI:** 10.3389/fcell.2019.00082

**Published:** 2019-05-31

**Authors:** Ning-Xin Ma, Jiu-Chao Yin, Gong Chen

**Affiliations:** Department of Biology, Huck Institutes of Life Sciences, Pennsylvania State University, University Park, PA, United States

**Keywords:** chemical reprogramming, transcriptome, astrocyte, neuron, signaling pathway

## Abstract

Chemical reprogramming of astrocytes into neurons represents a promising approach to regenerate new neurons for brain repair, but the underlying mechanisms driving this trans-differentiation process are not well understood. We have recently identified four small molecules – CHIR99021, DAPT, LDN193189, and SB431542 – that can efficiently reprogram cultured human fetal astrocytes into functional neurons. Here we employ the next generation of RNA-sequencing technology to investigate the transcriptome changes during the astrocyte-to-neuron (AtN) conversion process. We found that the four small molecules can rapidly activate the hedgehog signaling pathway while downregulating many glial genes such as FN1 and MYL9 within 24 h of treatment. Chemical reprogramming is mediated by several waves of differential gene expression, including upregulation of hedgehog, Wnt/β-catenin, and Notch signaling pathways, together with downregulation of TGF-β and JAK/STAT signaling pathways. Our gene network analyses reveal many well-connected hub genes such as repulsive guidance molecule A (RGMA), neuronatin (NNAT), neurogenin 2 (NEUROG2), NPTX2, MOXD1, JAG1, and GAP43, which may coordinate the chemical reprogramming process. Together, these findings provide critical insights into the molecular cascades triggered by a combination of small molecules that eventually leads to chemical conversion of astrocytes into neurons.

## Introduction

Adult brains have very limited regeneration capability. Therefore, direct neuronal reprogramming from non-neuronal cells provides a potential remedy for neuronal loss caused by neural injury or neurodegeneration. Ectopic expression of transcription factors (TFs) in non-neuronal cells has generated neural progenitors and neurons both *in vitro* and *in vivo* (Berninger et al., 2007; Heinrich et al., 2010; Grande et al., 2013; Mazzoni et al., 2013; Torper et al., 2013; Guo et al., 2014; Liu et al., 2015; Gascón et al., 2016; Wang et al., 2016). Subtype-specific neurons can also be induced by different combinations of TFs. For example, sustained expression of Neurog2 and Dlx2 in cortical astroglia can produce glutamatergic and GABAergic neurons, respectively (Heinrich et al., 2010, 2011). NeuroD1 can convert astrocytes into mainly glutamatergic neurons and convert NG2 cells into both glutamatergic and GABAergic neurons (Guo et al., 2014). In cell cultures, Ascl1, Nurr1, and Lmx1a can induce dopaminergic neurons from fibroblasts derived from Parkinson’s disease patients (Caiazzo et al., 2011). Another combination of NeuroD1, Ascl1, Lmx1a, and miR218 can produce dopaminergic neurons from astrocytes in an *in vivo* Parkinson’s disease mouse model (Rivetti Di Val Cervo et al., 2017). Besides TFs, small molecules have also been used to produce induced pluripotent stem cells (iPSCs) (Hou et al., 2013), neural progenitors (Cheng et al., 2014), neurons (Hu et al., 2015; Zhang et al., 2015; Gao et al., 2017), cardiomyocytes (Fu et al., 2015; Cao et al., 2016), and liver cells (Li et al., 2014) in *in vitro* cultures. Small molecules can be applied in combination with viral agents to improve the reprogramming efficiency (Chambers et al., 2009; Ladewig et al., 2012; Li et al., 2014; Pfisterer et al., 2016; Qi et al., 2017). Compared to viral-based delivery, chemical administration is easy to use and can be further developed into pharmaceuticals.

Studies of TF-mediated conversion have identified pioneer factors, binding events, transcriptional cascades, epigenetic modifications, and genetic networks that orchestrate the conversion process (Wapinski et al., 2013; Treutlein et al., 2016; Mall et al., 2017; Velasco et al., 2017). Small molecules have been found to improve the conversion efficiency through modulating chromatin accessibility (Smith et al., 2016) or increasing the TF binding (Abad et al., 2017). Chemicals alone can also activate endogenous Sox2 through the bFGF and Shh pathway to generate neural stem cells from fibroblasts (Zhang M. et al., 2016). In our previous work, we have identified a cocktail of nine small molecules that can directly convert human astrocytes (HAs) into neurons in cell cultures (Zhang et al., 2015). Our following work further identified a four-molecule combination that can also chemically reprogram HAs directly into neurons (Yin et al., 2019). However, the molecular mechanisms underlying such efficient chemical reprogramming from an astrocyte into a neuron are not well understood.

In this study, we employed RNA-sequencing technology to investigate the transcriptome dynamics in cultured HAs during the chemical conversion process toward neuronal phenotype. We identified several specific waves of gene expression that might be critical in different stages during the chemical conversion process. It started with strong suppression on the TGF-β and JAK/STAT signaling pathway, forcing the astrocytes out of the cell cycle. At the same time, activation of hedgehog and Wnt/β-catenin signaling pathways led to the second wave of gene expression on the neurogenic network. Besides the well-known bHLH TFs (NGN, NeuroD, ASCL), we also identified several new components that were involved in chemical conversion, such as the gene expression of neuronatin (NNAT), jagged canonical notch ligand 1 (JAG1), and repulsive guidance molecule A (RGMA). We observed concurrent changes in metabolism and extracellular matrix but not in progenitor properties. Neuronal identity was confirmed by forebrain-specific markers, and genes related to axon guidance and synaptic function. Our transcriptome analyses provide mechanistic insights into how neuronal identities are acquired from a non-neuronal cell with a chemical modulation approach, which may provide a blueprint for developing chemical therapies for brain repair.

## Results

### Genome-Wide Profiling of Chemically Induced Astrocyte-to-Neuron Conversion

**FIGURE 1:**
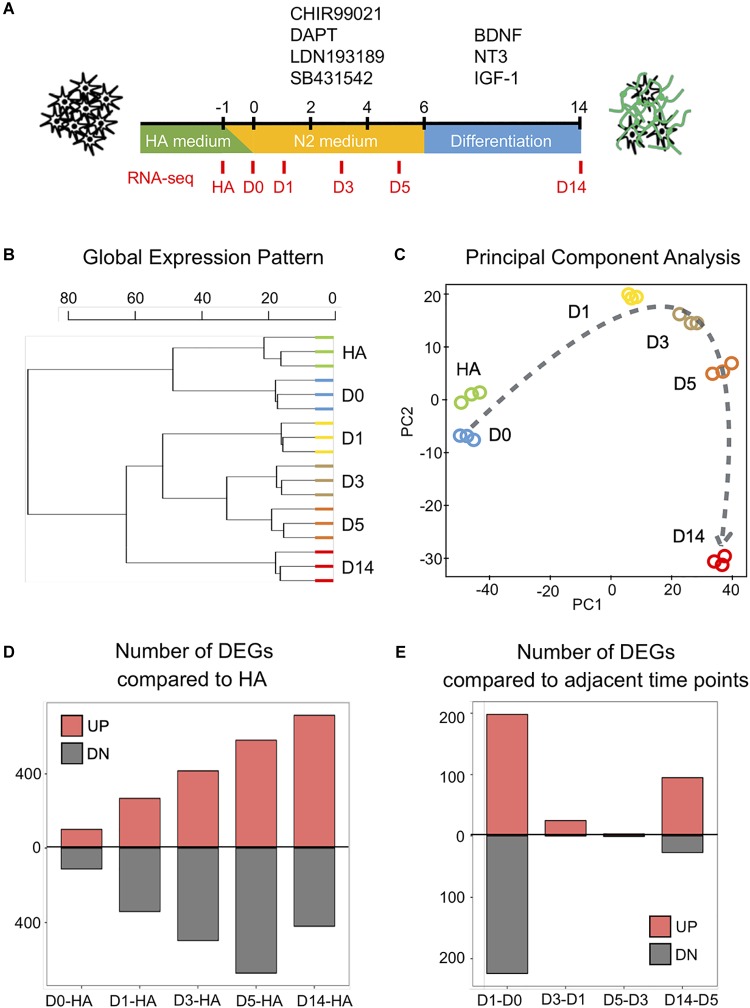
Genome-wide changes in response to the core drug treatment. **(A)** Schematic illustration of the overall experimental design. The four core drugs (CHIR99021, DAPT, LDN193189, SB431542) were added on day 0 and refreshed every other day in N2 medium. After 6 days, cells were transferred into differentiation medium with neurotrophic factors to promote for neuronal maturation. **(B)** Hierarchical clustering among all the samples from human astrocytes (HAs), day 0 (D0), day 1 (D1), day 3 (D3), day 5 (D5), and day 14 (D14) based on the expression of 16,324 genes detected (*n* = 3 biological replicates for each time point). **(C)** Principal component analysis of all the samples. Dashed line indicates the conversion trajectory. Note that HA and D0 samples were before drug treatment, D1–D5 samples were during drug treatment, and D14 was after drug treatment. **(D)** Histogram shows the number of differentially expressed genes (DEGs) (adjusted *p* < 0.01, fold change >3) among D0–D14 samples in all the pair-wise comparisons with HA. **(E)** Histogram of the number of DEGs in pair-wise comparisons between adjacent time points (D1–D0, D3–D1, D5–D3, D14–D5).

Previously, we have demonstrated that efficient astrocyte-to-neuron (AtN) conversion can be accomplished by treatment with a cocktail of nine small molecules (Zhang et al., 2015). In an effort to simplify the chemical reprogramming recipe, we further identified four core drugs, CHIR99021, DAPT, LDN193189, and SB431542, that can be added together to reprogram HAs into neurons (Yin et al., 2019). CHIR99021 is a selective glycogen synthase kinase 3 (GSK-3) inhibitor and can activate the Wnt/β-catenin pathway in human and mouse stem cells (Wu et al., 2013; Nadar et al., 2015). DAPT can inhibit γ-secretase and Notch signaling and has been used to promote neuronal differentiation in ES cells (Crawford and Roelink, 2007; Borghese et al., 2010; Qi et al., 2017). LDN193189 is an inhibitor of bone morphogenetic protein (BMP) type I receptors ALK2 and ALK3 and has been used to suppress mesoderm and endoderm specification (Zhang M. et al., 2016). SB431542 inhibits TGF-β type I receptors ALK4, ALK5, and ALK7. These four drugs were added simultaneously into a culture medium with N2 supplement on day 0 and refreshed every 2 days. After 6 days, all the chemicals were withdrawn, and N2 medium was changed to a differentiation medium containing serum, neurotrophic factors, and growth factors brain-derived neurotrophic factor (BDNF), neurotrophin-3 (NT3), insulin-like growth factor 1 (IGF-1) to facilitate neuronal growth and maturation (see Figure 1A for schematic illustration). These four molecules were found to efficiently convert HAs into fully functional neurons, which could survive > 7 months in culture (Yin et al., 2019).

To understand the molecular mechanisms underlying the four-molecule-mediated chemical reprogramming, we investigated the transcriptome changes during chemical treatment using deep RNA-sequencing (RNA-seq) technology. Human fetal astrocytes (HA1800, ScienCell) were used for chemical reprogramming because they have been widely used in the field. Three independent mRNA samples (biological replicates) were collected from each of the six time points (HA, days 0, 1, 3, 5, and 14), corresponding to before (HA, day 0), during (days 1, 3, 5), and after (day 14) chemical treatment (Figure 1A). A box plot of the raw data (Supplementary Figure S1A, represented by the distribution of log2 read counts) suggests that despite minor differences between the medians, comparable data distributions were found among all the samples. On day 14, the reprogramming efficiency was characterized by immunostaining of neuronal markers NeuN and MAP2, and the quantification results confirmed that around 60% of the cells were NeuN-positive neurons (59.4% ± 1.8%, mean ± SEM) (Supplementary Figures S1C,D). Principal component analysis (PCA) analysis of our HA samples and D14 samples together with previously reported data sets, including chemically induced 30 days neuron (GSE84826), iPSC-derived 30 days neuron (GSE102352), iPSC-derived 7 weeks neuron (GSE88773), and 25 years cortex samples (GSE73721), indicates that our D14 neurons are closer to other neuronal profiles but distant from the HA profile (Supplementary Figure S1E).

When comparing the global gene expression pattern among our different samples, the dendrogram plot showed a clear shift of the overall gene profile before and after chemical treatment (Figure 1B). Specifically, HA and day 0 samples before drug treatment showed similar gene profiles, while day 14 samples after drug treatment showed a different profile compared to days 1–5 samples during drug treatment (Figure 1B). Similarly, the principal component analysis also showed that samples closer in time were mapped closer on the plot, and the core drugs steadily pushed astrocytes through a conversion trajectory (Figure 1C).

Next, we performed pair-wise differential expression analysis at different time points of chemical treatment. A total of 1,889 differentially expressed genes (DEGs) were identified (fold change > 3, read mean > 80, adjusted *p* < 0.01). The overall pair-wise gene expression comparison between different samples is shown in Supplementary Figure S2. Figure 1D shows that compared to the HA samples, the number of upregulated and downregulated DEGs showed a steady increase along with the core drug treatment. However, when samples from adjacent time points were compared (Figure 1E), the most dramatic change occurred within the first 24 h of drug treatment. Therefore, small molecule treatment induced a rapid transcriptome change in HAs within the first day of chemical reprogramming.

**FIGURE 2:**
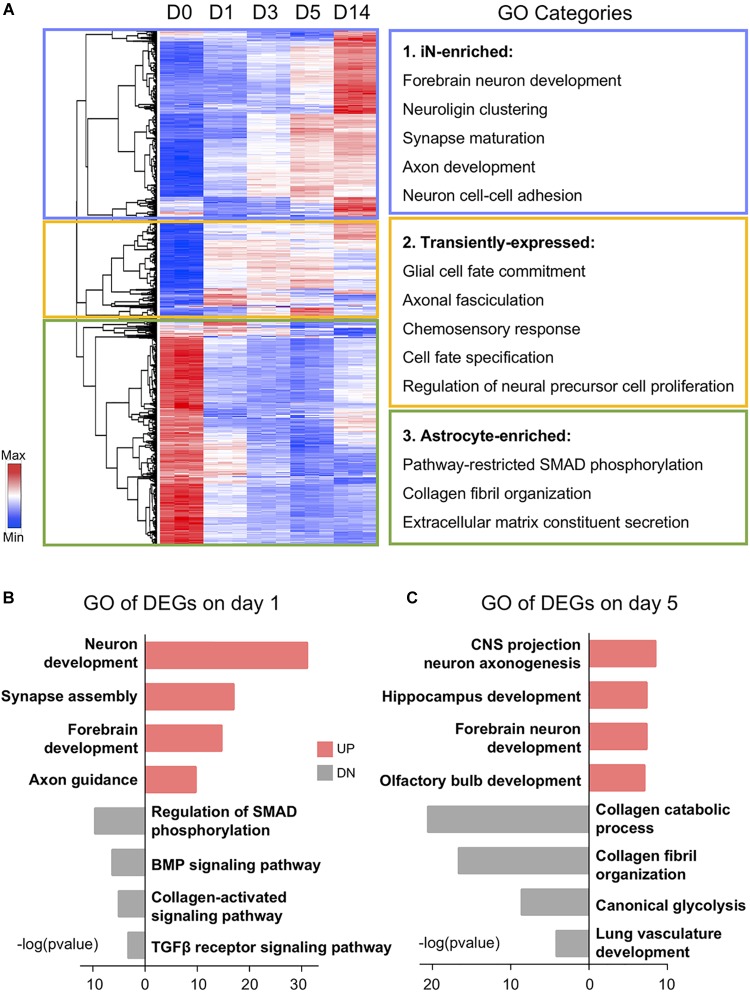
Gene ontology (GO) analysis showing functional transition from astrocytes to neurons. **(A)** Hierarchical clustering and heat map of RNA-seq data showing all the differentially expressed genes (1,889 DEGs) among D0–D14 samples. Red color indicates high expression level, whereas blue color indicates low expression level. DEGs were divided into three clusters based on their expression patterns. GO categories associated with each cluster are shown on the right. **(B,C)** Top four significant GO terms associated with upregulated (UP) and downregulated (DN) genes on days 1 and 5 of small molecule treatment. Note that upregulated GO terms are associated with neuronal genes and downregulated GO terms are associated with glial genes.

After analyzing the overall changes of the DEG profile, we looked into the detailed categories of the DEGs during chemical reprogramming. Using a hierarchical clustering method, all 1,889 DEGs could be classified into three clusters based on their expression patterns: (1) neuron-enriched genes (Figure 2A, blue box); (2) transitional genes (Figure 2A, yellow box); and (3) astrocyte-enriched genes (Figure 2A, green box). This first cluster includes 698 genes enriched in day 14 induced neurons (iNs), which were initially very low in HAs but gradually increased during chemical treatment. The gene ontology (GO) terms associated with this first gene cluster include forebrain neuron development, neuron cell–cell adhesion (including neuroligin cluster), axon development, and synapse maturation. The second cluster has 353 DEGs that were also initially low in astrocytes but then activated during core drug treatment. The GO terms associated with this group are chemosensory response, axonal fasciculation, and cell fate commitment and specification, suggesting a molecular activation of cell fate change. The third cluster of the remaining 838 genes was highly expressed in day 0 astrocytes but significantly downregulated after small molecule treatment, which involved biological processes including collagen fibril organization, extracellular matrix constituent secretion, and SMAD phosphorylation.

Besides the gradual shift of gene expression pattern, DEGs at different time points were also involved in diverse biological processes (Figure 2B,C). For example, many of the downregulated genes on day 1 after chemical treatment are associated with SMAD phosphorylation, BMP signaling, and TGF-β receptor signaling (Figure 2B). This is expected because we used a BMP receptor inhibitor LDN193189 and a TGF-β receptor inhibitor SB431542. These two chemicals together can have a dual inhibition on the SMAD pathway, which has been shown to potentiate the neural conversion of fibroblasts (Chambers et al., 2009; Ladewig et al., 2012; Liu et al., 2013; Pfisterer et al., 2016). Moreover, along the chemical treatment at day 5, regional neurogenesis such as hippocampus development and olfactory bulb development emerged after general forebrain neuron development (Figure 2C). In the meantime, astrocyte-related functions, including glycolysis and collagen metabolism and organization, have been suppressed by the drug treatment at day 5 (Figure 2C). Together, these results indicate an upregulation of neuronal GO terms accompanied by a downregulation of astroglial GO terms during chemical treatment.

### Suppression of Astroglial Genes and Activation of Neuronal Genes by Small Molecules

**FIGURE 3:**
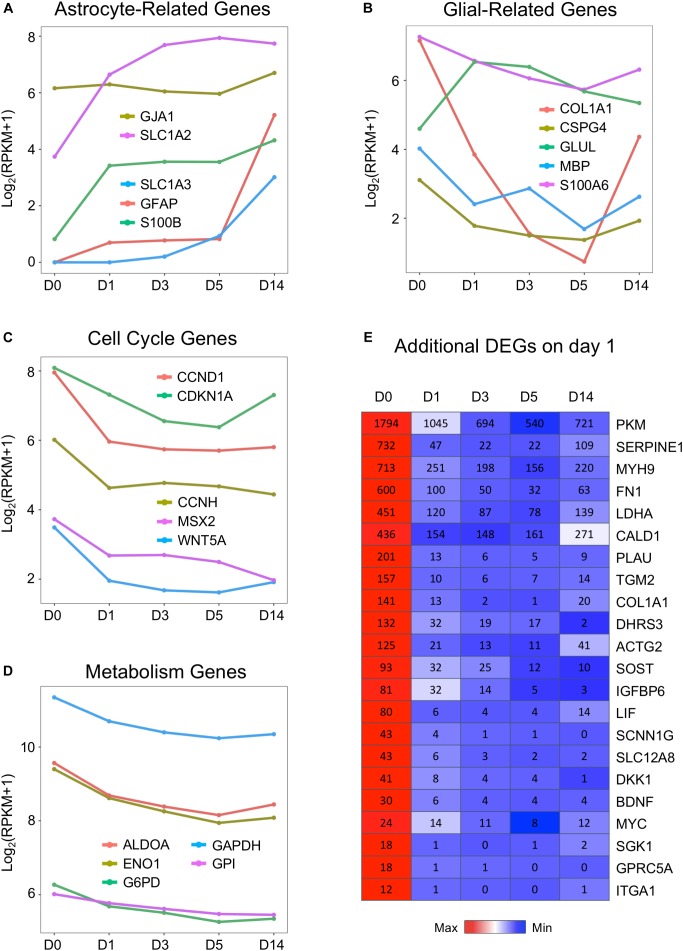
Downregulation of cell cycle and metabolic genes by small molecules. **(A,B)** Representative glial marker genes during chemical reprogramming process. The small molecule treatment did not change the transcriptional level of GFAP and S100B. The glutamate transporters (SLC1A2 or EAA2/GLT-1 and SLC1A3 or EAA1/GLAST) were upregulated, whereas COL1A1 (collagen) and CSPG4 (NG2) were downregulated. **(C)** Genes related to the cell cycle process showed decreased expression, with a significant drop at D1. **(D)** Genes involved in glycolysis (ALDOA, ENO1, GAPDH, G6PD) were uniformly downregulated by small molecule treatment. **(E)** Heat map of representative genes showing significant downregulation during the chemical conversion process. Color scaled within each row and FPKM values are presented. Red color indicates highest expression level within each row, whereas blue color indicates low expression level within each row.

Following the GO analysis, we further investigated some individual genes among the DEGs and analyzed their pattern of changes before, during, and after chemical treatment. In particular, because our chemical reprogramming method converted HAs into neurons, we therefore looked into how individual glial genes and neuronal genes were affected during the chemical conversion process. Interestingly, we found that the expression patterns among several typical astrocyte-enriched genes were not uniformly changed. For example, the widely used astrocytic markers GFAP and S100B increased on day 1 but then stayed steady at day 3 and day 5 (Figure 3A, red line and green line). The increase at day 14 is likely caused by the withdrawal of small molecules at day 6, resulting in a proliferation of astrocytes (Yin et al., 2019). Glutamate transporter GLT-1 (also known as SLC1A2) is highly expressed in the brain and serves as a major transporter responsible for glutamate uptake (Matsugami et al., 2006). It is interesting to observe a significant increase of GLT-1 during small molecule treatment (Figure 3A, purple line), consistent with the notion that GLT-1 is expressed not only in astrocytes but also in neurons (Brooks-Kayal et al., 1998; Mennerick et al., 1998). Another astrocytic glutamate transporter GLAST (SLC1A3) is expressed at a low level in HAs but also highly upregulated during drug treatment (Figure 3A, blue line). On the other hand, other glia-related genes such as type I collagen (COL1A1), myelin basic protein (MBP), chondroitin sulfate proteoglycan 4 (CSPG4), and S100A6 gradually decreased in response to core drug treatment (Figure 3B). The rebound of COL1A1 on day 14 is also related to the astrocytic proliferation after removal of the drugs. Importantly, our drug treatment strongly inhibited astroglial proliferation, as shown by consistent downregulation of cell cycle genes including cyclin-D (CCND1), cyclin-H (CCNH), and cyclin-dependent kinase inhibitor 1A (CDKN1A) (Figure 3C). The inhibition of cell proliferation by small molecule treatment at D1 suggests that astrocytes are not turning into neural progenitors.

Astrocytes are known to have a high glycolytic rate, while neurons rely more on oxidative phosphorylation (Bélanger et al., 2011; Zheng et al., 2016). We found that one category of astrocyte-enriched genes that showed uniform downregulation was closely associated with metabolism (Figure 3D). For example, GAPDH (glyceraldehyde 3-phosphate dehydrogenase, an important enzyme for glycolysis), aldolase A (also known as fructose-bisphosphate aldolase, a glycolytic enzyme), and G6PD (glucose-6-phosphate dehydrogenase) were all downregulated during small molecule treatment (Figure 3D). In addition, lactate dehydrogenase (LDHA) and pyruvate kinase M (PKM) were both significantly decreased during drug treatment (Figure 3E). In fact, among the most significantly downregulated genes, many were glycolysis-related genes (Figure 3E). Other highly downregulated genes included some cytoskeleton and extracellular components, such as myosin (MYH9), fibronectin (FN1), actin (ACTG2), caldesmon (CALD1), and collagen (COL1A1). These results suggest that many glia-related genes were suppressed by our core drug treatment.

**FIGURE 4:**
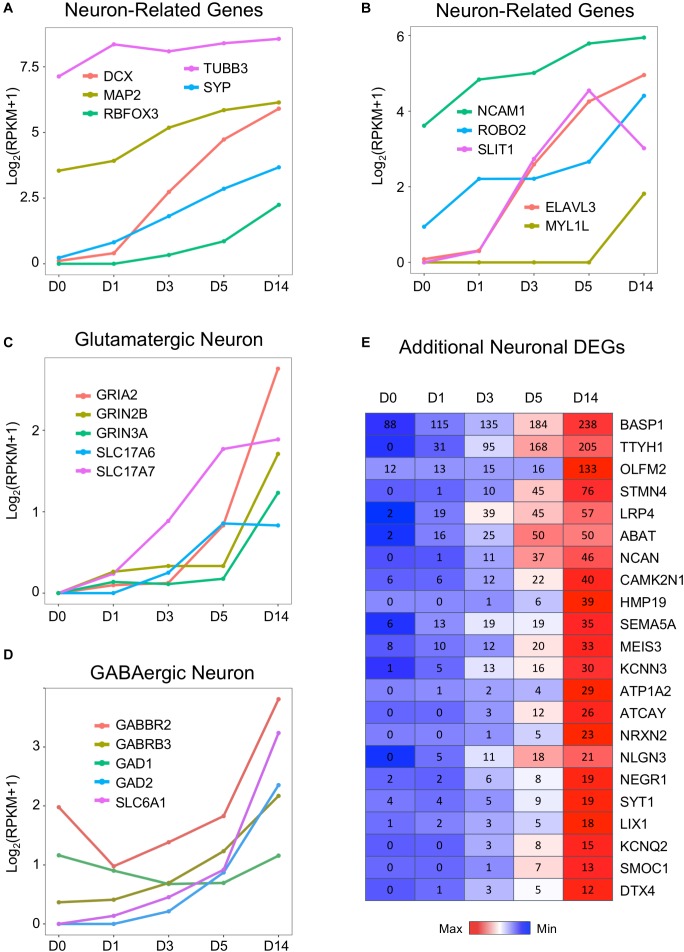
Neuronal genes were activated by the core drugs and highly expressed in D14 samples. **(A)** Typical neuronal marker genes such as doublecortin (DCX), RBFOX3 (NeuN), MAP2, and synaptophysin (SYP) were highly upregulated upon the application of small molecules. Note that DCX increased much faster than NeuN. Interestingly, βIII-tubulin (TUBB3, often labeled with TUJ1 antibody) was highly expressed in HAs and only modestly increased during chemical treatment. **(B)** Neuronal cell adhesion molecules (NCAM1) and chemoattractant/chemorepellent genes (SLIT1/ROBO2) were upregulated by small molecule treatment. **(C)** Dramatic increase of glutamatergic neuron–related genes, including subunits of AMPA receptors (GRIA) and NMDA receptors (GRIN), as well as vesicular glutamate transporters (SLC17A6 = VGLUT2; SLC17A7 = VGLUT1). **(D)** Expression profile of GABAergic neuron–related genes, including GABA receptor subunits, glutamate decarboxylases, and GABA transporters. GABBR2 = GABA type B receptor subunit 2. GABRB3 = GABA type A receptor subunit β3. GAD1 = GAD67. GAD2 = GAD65. SLC6A1 = GAT1. **(E)** Heat map of representative genes that were highly upregulated in D14 samples after core drug treatment. Color scaled within each row and FPKM values presented.

In contrast to the downregulation of glial genes, many neuronal genes are upregulated during chemical conversion (Figure 4). For example, immature neuron marker DCX, neuronal nuclei marker RBFOX3 (NeuN), and synaptic vesicle development marker SYP were barely expressed in astrocytes (D0) but considerably increased during chemical treatment (D1–D5) and continued to increase in D14 neurons (Figure 4A). Similarly, genes related to axon guidance (ROBO2, SLIT1) and a neural-specific RNA-binding protein ELAVL3 were initially low in astrocytes but highly upregulated by small molecule treatment (Figure 4B). The neural TF MYT1L (Mall et al., 2017) was almost undetectable from D0 to D5 samples and only showed significant expression at D14 (Figure 4B). Interestingly, a neural adhesion molecule NCAM1 (Cremer et al., 2000) and a mature neuron marker MAP2 were modestly expressed in HAs and then upregulated during chemical reprogramming (Figure 4A,B). A surprising finding is that compared to other neuron-specific genes, the class III β-tubulin (TUBB3) was expressed at a high level in HAs (Figure 4A), with only a modest increase after chemical conversion. Such high expression of TUBB3 might be related to the fetal origin of our HA cultures, which can be different from adult HAs (Yin et al., 2019). Nevertheless, the drastic difference between the expression level of TUBB3 versus DCX and NeuN in HAs suggests that TUBB3 should be used in combination with other neuronal markers such as NeuN and MAP2 to determine neuronal identity (Dráberová et al., 2008).

Using immunostaining and electrophysiological recordings, we have characterized the small molecule–converted neurons as mainly glutamatergic neurons (78%) (Yin et al., 2019). Consistently, our RNA-seq data confirmed the upregulation of glutamate receptors (including both AMPA and NMDA receptor subunits GRIA2, GRIN2B, and GRIN3A), as well as vesicular glutamate transporters SLC17A6 (VGLUT2) and SLC17A7 (VGLUT1), during and after chemical reprogramming (Figure 4C). Interestingly, despite the fact that GABAergic neurons only accounted for a small proportion (2%) in our chemically reprogrammed neurons, GABAergic neuron–related genes were also upregulated, such as the GABA receptor subunit GABBR2 and GABRB3, glutamate decarboxylase (GAD1, GAD2), and GABA transporter SLC6A1 (also known as GAT1) (Figure 4D). It is interesting to note that there is a significant expression level of GABBR2 in HAs (Figure 4D). Whether HAs can respond to GABA stimulation is worth of further exploring.

Besides the aforementioned common neuronal genes, the heat map in Figure 4E further lists some genes that are very low or undetectable in HAs (D0) but then upregulated in D14 neurons after small molecule treatment. These genes include a possible chloride anion channel TTYH1; a Ca^2+^-binding protein SPARCL1; a protein tyrosine phosphatase PTPRZ1; as well as cortical neuron markers DTX4, CUX2, and LIX1 (Molyneaux et al., 2007; Figure 4E). Synaptic cell adhesion molecules such as neuroligin (e.g., NLGN3) (Budreck and Scheiffele, 2007) and neurexin (e.g., NRXN2), as well as neuron-specific matrix protein HMP19 were also upregulated after chemical conversion. Together, we conclude that our small molecule treatment significantly suppresses astroglial genes but highly upregulates neuronal genes.

### Activation of bHLH Transcription Factors During Chemical Reprogramming

**FIGURE 5:**
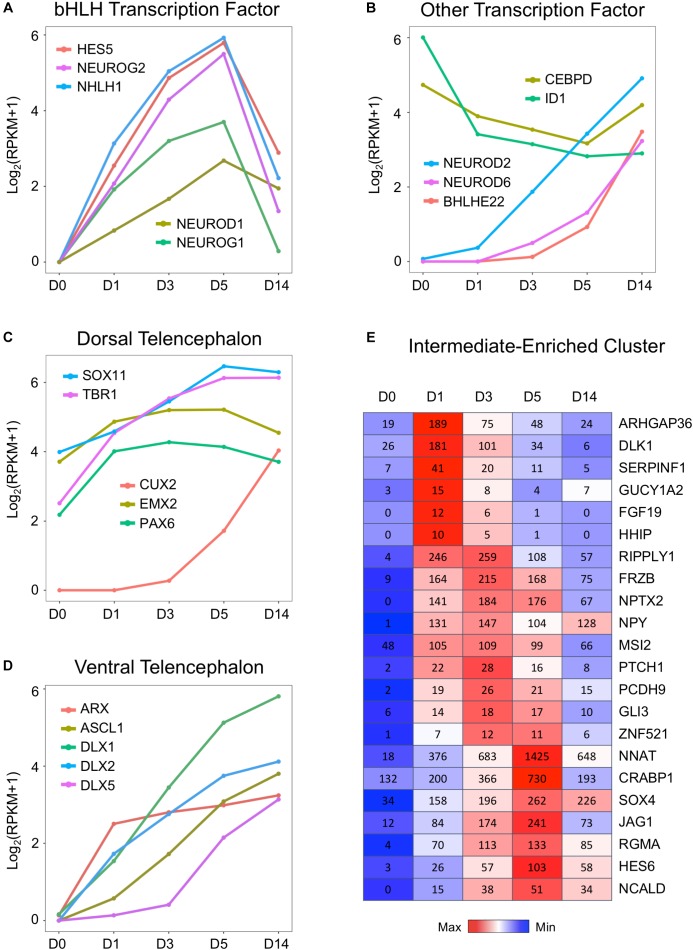
Upregulation of neural transcription factors during chemical reprogramming of astrocytes into neurons. **(A)** Rapid activation of the bHLH family of transcription factors involved in neurogenic development process during the chemical conversion process. Note that these neural transcription factors were downregulated at D14 when neuronal conversion was completed. **(B)** Additional neural transcription factors that were upregulated by small molecules, such as NEUROD2/6 and BHLEH22. In contrast, DNA binding inhibitor ID1 and astrocytic enhancer binding CEBPD were downregulated. **(C)** Genes involved in dorsal telencephalon development were upregulated during core drug treatment. Note that cortical neuron marker genes TBR1 and CUX2 were significantly increased by small molecules. **(D)** Transcription factors involved in ventral telencephalon development such as ASCL1 and its downstream targets ARX and DLX were also upregulated. **(E)** Representative gene expression waves that peaked at D1, D3, and D5 during the chemical conversion process. The first wave peaked on D1 included several hedgehog genes such as ARHGAP36 and DLK1. Color scaled within row and FPKM values presented.

What triggered the activation of neuronal genes in an astroglial cell? To answer this question, we investigated a family of basic helix–loop–helix (bHLH) TFs that is well known to play an important role in neural differentiation during early brain development (Parras et al., 2002; Ross et al., 2003; Roybon et al., 2010). Figure 5A illustrates a uniform and continuous increase of gene expression level of several bHLH factors NEUROD1, NEUROG1, NEUROG2, HES5, and NHLH1 during chemical treatment from D1 to D5, followed by rapid downregulation at D14 after completion of AtN conversion. The RNA-seq analysis here confirmed our recent finding using qRT-PCR analysis that both NEUROD1 and NEUROG2 were significantly upregulated by core drug treatment (Yin et al., 2019). Other TFs were further upregulated at D14 after being turned on by core drugs (Figure 5B), such as BHLHE22/BHLHB5, the TF for layer II–V neurons (Joshi et al., 2008), as well as NEUROD2 and NEUROD6, which are key regulators of axonogenesis and fasciculation (Bormuth et al., 2013). In contrast, the anti-bHLH protein ID1 was initially expressed very highly in HAs at D0 but significantly downregulated by small molecule treatment (Figure 5B). Similarly, astrocytic enhancer binding protein CEBPD (Ko et al., 2014) also showed a significant downregulation during chemical treatment (Figure 5B). These results suggest that our core drug-induced AtN conversion process partially recapitulates some aspects of neurodevelopment, where bHLH TFs are activated to control neuronal fate specification.

Consistent with our observation that the majority of core drug-converted cells were glutamatergic cortical neurons (Yin et al., 2019), our transcriptome analysis revealed an activation of multiple TFs involved in the dorsal telencephalon development, including PAX6, EMX2, CUX2, SOX11, and TBR1 (Warren et al., 1999; Schuurmans and Guillemot, 2002; Wang et al., 2013). PAX6 and TBR1 are sequentially expressed in the developing neocortex where radial glia differentiate into neurons (Englund, 2005). EMX2 is a direct target of Wnt and BMP (Theil et al., 2002), and CUX2 is a downstream target of Notch (Iulianella et al., 2009). Interestingly, besides dorsal TFs, the ventral TFs were also upregulated (Figure 5D). For example, members of distal-less family DLX1/2/5 are the downstream targets of proneural TF ASCL1, and they all showed a significant upregulation after chemical treatment (Figure 5D). The DLX factors can regulate the generation of GABAergic neurons and drive the expression of ARX, GAD1, and GAD2 (Colasante et al., 2008; Le et al., 2017; Yang et al., 2017). Note that compared to the relatively high expression level of dorsal TFs such as Sox11, EMX2, TBR1, and Pax6 in our cultured human cortical astrocytes at D0 (Figure 5C), the ventral TFs (Figure 5D) were almost undetectable in our HAs at D0, confirming the origin of our astrocytes from cortical tissue. We also investigated several neural progenitor markers such as NES and SOX2 during chemical reprogramming, which only showed an increase at D1 but then remained relatively flat throughout D3–D14 (Supplementary Figure S1B). One interesting finding is that vimentin is highly expressed in HAs and not affected by small molecule treatment (Supplementary Figure S1B). Together with a decrease in cell cycle–related genes during chemical treatment (Figure 3C), these results suggest that during our chemical reprogramming, the astrocytes did not de-differentiate into neural progenitors.

We further investigated the transiently expressed gene clusters during chemical treatment at D1–D5 and found several waves of gene expression (Figure 5E). The first wave of genes that were rapidly activated by core drug administration and reached peak expression level at D1 included a non-canonical Notch ligand DLK1 (Falix et al., 2012) and ARHGAP36, a Rho GTPase–activating protein that activates hedgehog but antagonizes PKA, as well as a hedgehog gene FGF19. When the first wave of gene expression receded, the second wave of gene expression peaked at D3, including a transcriptional repressor RIPPLY1, a Wnt-binding protein FRZB, and NPTX2, a neuronal pentraxin protein that plays an important role in synapse formation (Figure 5E). The third wave of gene expression that was activated by small molecule treatment at D1 and peaked at D5 before receding at D14 includes NNAT, a lipoprotein playing an important role during brain development through regulating ion channels, and CRABP1, a retinoic acid binding protein, as well as JAG1, a cell surface protein interacting with the Notch signaling pathway. These sequentially expressed genes together with many other genes may trigger signaling cascades, initiate neurogenesis, and gradually push the astrocytes to adopt a neuronal identity.

### Upregulation and Downregulation of Signaling Pathways

**FIGURE 6:**
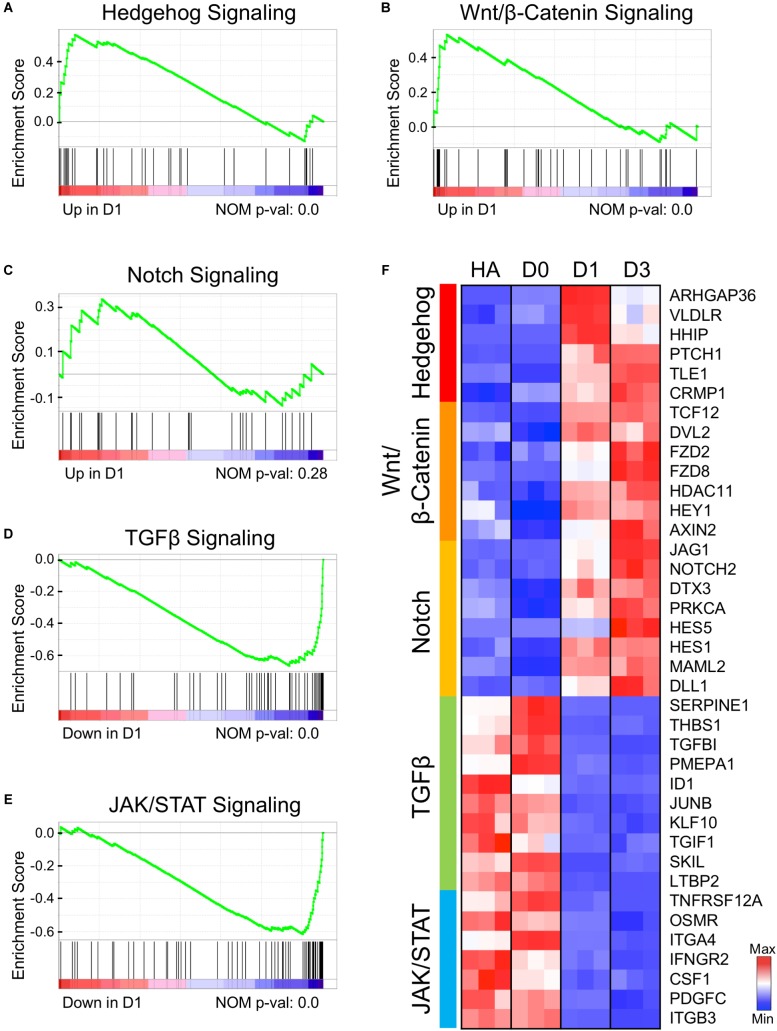
Early genetic alternations of signaling pathways in response to core drug treatment. **(A–E)** Gene set enrichment analysis (GSEA) of D1 samples compared to HAs revealed several signaling pathways that were either activated **(A–C)** or suppressed **(D,E)** at the initiation stage of chemical reprogramming. **(F)** Heat map illustrating the leading-edge subsets of genes corresponding to each signaling pathway shown in **(A–E)**. Color scaled within each row. Red color indicates high expression level, while blue color indicates low expression level.

By substituting the four core drugs with their functional analogs, we have demonstrated that modulating GSK-3β, Notch, BMP, and TGF-β signaling pathways is critical for chemical reprogramming (Yin et al., 2019). Here, we investigated the transcriptional profile related to these four signaling pathways at the initiation stage of chemical reprogramming. The gene set enrichment analysis (GSEA) on D1 samples revealed an upregulation of hedgehog, Wnt/β-catenin, and Notch signaling pathways (Figure 6A–C), and a downregulation of TGF-β and JAK/STAT3 signaling pathways (Figure 6D,E). The heat map of Figure 6F shows the leading-edge subsets of genes from each signaling pathway. Remarkably, the hedgehog signaling pathway was turned on immediately at D1 after small molecule treatment (Figure 6F, top cluster), which included ARHGAP36, VLDL4, HHIP, and PTCH1. ARHGAP36 is a positive regulator of hedgehog and an inhibitor of protein kinase A (PKA) (Eccles et al., 2016). The hedgehog–PKA pathway is responsible for cell cycle exit and neuronal differentiation (Masai et al., 2005). The upregulation of ARHGAP36 at D1 suggests that the antagonistic effect between hedgehog and PKA may play an important role in AtN conversion as well. Following the rapid activation of hedgehog signaling, the Wnt/β-catenin and Notch signaling pathways were also upregulated at D1 and further enhanced at D3 (Figure 6F). For Wnt pathway, the Disheveled 2 (DVL2) is an activator of Wnt, and frizzled family members FZD2/FZD8 encode receptors for Wnt proteins (Figure 6F; Huang and Klein, 2004; Smalley, 2005). At the same time, activation of Wnt signaling also induced its negative regulator AXIN2 (Jho et al., 2002). The upregulation of Notch pathway includes NOTCH2 and NOTCH family ligands JAG1 and DLL1, HES family members HES1/HES5, and the cytoplasmic regulator deltex-3 (DTX3). Notch family members have been well known for their functions in neural differentiation (Fathi et al., 2011). While the upregulation of Wnt/β-catenin signaling is expected because of CHIR99021 inhibition of GSK-3β and activation of Wnt/β-catenin signaling, the upregulation of Notch signaling is unexpected because of the Notch inhibitor DAPT used in our core drug treatment. Paradoxically, removing DAPT from the four core drugs resulted in a significant decrease of neuronal conversion efficiency, as shown in our recent published work (Yin et al., 2019). We hypothesize that although DAPT directly inhibits Notch signaling, when combined with other signaling modulators together, the Notch pathway might be transiently reversed to an active state, but the precise mechanisms certainly warrant further investigation.

Among the downregulated TGF-β signaling factors, SERPINE1 (PAI-1) is an inhibitor of serine protease, and TGFBI encodes an extracellular matrix protein. Both genes are TGF-β targets and usually express in astrocytes after injury (Docagne et al., 2002; Logan et al., 2013). Another decreased gene is thrombospondin-1 (THBS1), an adhesive glycoprotein regulated by TGF-β, EGF, and STAT3. THBS1 is responsive to hypoxia injury and can act as a synaptogenic molecule (Christopherson et al., 2005; Mense et al., 2006; Tyzack et al., 2014). The significantly downregulated factors in the JAK/STAT pathway include a TWEAK receptor TNFRSF12A and a cytokine CSF1, both of which respond to inflammation and astrogliosis (Zamanian et al., 2012; Khawar et al., 2016). Another receptor from the IL-6 family, OSMR (oncostatin M receptor), is known to be involved in STAT3 activation and GFAP expression in astrocyte differentiation (Yanagisawa et al., 1999). Other downregulated genes include an interferon-γ receptor IFNGR2, integrin subunits ITGA/B, and PDGFC, which can de-differentiate astrocytes in glioma cell lines (di Tomaso et al., 2009). In addition, we also found that the extracellular matrix–related genes such as collagen and fibronectin signaling pathways were significantly downregulated (Supplementary Figure S3). Together, the downregulation of TGF-β and JAK/STAT3 signaling factors indicates the loss of astroglial properties after small molecule treatment.

### Gene Network Analyses Reveal Well-Connected Hub Genes

**FIGURE 7:**
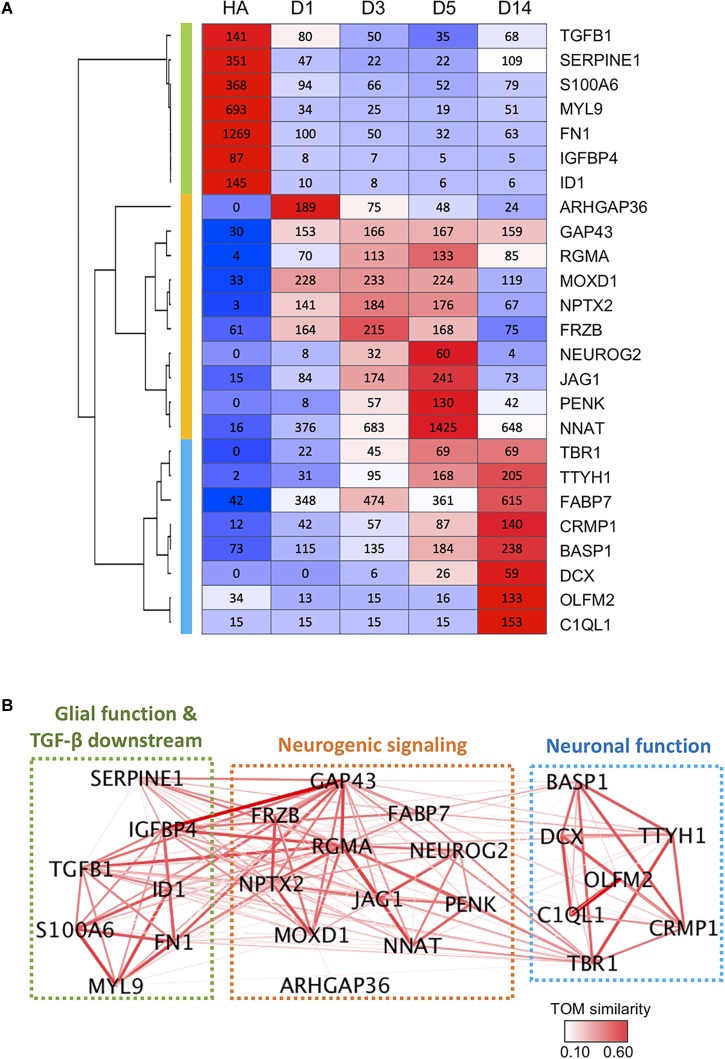
Gene co-expression network during chemical reprogramming. **(A)** Top 25 most highly expressed DEGs with significant changes during chemical reprogramming process. Three major clusters were identified and are color-coded on the left bar (green, orange, and blue), which correspond to the color squares in **(B)**. **(B)** The weighted correlation network of genes shown in panel A was plotted using WGCNA. Edge color and thickness indicate TOM similarity. Different clusters had been grouped together in three squares, and their functional connections were annotated. See also Supplementary Figure S4 for weighted degree of connectivity (node size) plotted by igraph.

Finally, we constructed a gene network based on pair-wise correlation of the top 25 DEGs that were both highly expressed and highly regulated during the chemical reprogramming process. These genes experienced the most dramatic changes and had high expression levels at peak time point (peak level > 50 RPKM). These top 25 DEGs can be categorized into three functional clusters (Figure 7A,B), including glial/TGF-β signaling genes (green cluster), neurogenic signaling genes (orange cluster), and neuronal function–related genes (blue cluster). For downregulated genes, glial genes fibronectin (FN1) and myosin light chain (MYL9) were highly expressed in astrocytes (Liesi et al., 1986; Tiu et al., 2000) but dramatically decreased by more than 10-fold at D1 after small molecule treatment (Figure 7A). Among the upregulated genes, ARHGAP36, RGMA, NPTX2, NEUROG2, PENK, TBR1, TTYH1, and DCX were expressed at a very low level or almost undetectable in HAs but were activated to a high level after small molecule treatment (Figure 7A). Another gene worth mentioning is NNAT, which has been reported to promote neuronal fate and regulate synaptic plasticity (Lin et al., 2010; Oyang et al., 2011). We found that NNAT was upregulated by > 20-fold at D1 after small molecule treatment and further increased by another fourfold from D1 to D5, making it one of the most highly expressed genes (1,425 RPKM) among all the DEGs (Figure 7A). Even at D14, NNAT remained highly expressed (648 RPKM), suggesting an important role of NNAT not only during AtN conversion but also during the following neuronal maturation process.

Figure 7B illustrates the gene network based on pair-wise correlation. Many genes were identified as a hub to link many other DEGs. The highly connected hub genes include RGMA, GAP43, TBR1, IGFBP4, TGFB1, NNAT, NPTX2, FN1, MOXD1, SERPINE1, ID1, FABP7, and TTYH1 (Figure 7B and Supplementary Figure S4). One striking finding is that glial/TGF-β–related genes (green cluster) are closely connected with neurogenic genes (orange cluster), as shown by thick line connections (Figure 7B). Among the hub genes, the RGMA, a BMP co-receptor that activates the SMAD signaling pathway, appeared to play a central role in coordinating neurogenic genes as well as glial genes (Figure 7B). GAP-43, a growth-associated gene known for regulating neurite outgrowth (Korshunova et al., 2008), was also found to be connected to glial genes such as IGFBP1, SERPINE1, TGFB1, ID1, and FN1 (Figure 7B). TBR1, on the other hand, appeared to be an important coordinator connecting to both neurogenic genes and neuronal function genes (Figure 7B).

Together, these gene network analyses suggest that chemical reprogramming is mediated through a concerted regulation of both glial and neurogenic genes.

## Discussion

In this work, we employed deep RNA-sequencing technology to analyze the transcriptomic changes during small molecule–mediated AtN conversion. The four chemicals CHIR99021, DAPT, LDN193189, and SB431542, when added together, can significantly alter gene expression level among multiple signaling pathways such as GSK-3β/Wnt/β-catenin, Notch, and TGF-β/SMAD. Other signaling pathways like hedgehog and JAK/STAT are also significantly modified. These signaling changes lead to the activation of the neurogenic TF network, including the members of the bHLH family. Meanwhile, the transcriptome change from astrocytes to neurons was accompanied by a metabolic transition from glycolysis to oxidative phosphorylation, together with a reduced proliferation rate. Our gene network analyses further revealed a close relationship between neurogenic genes and glial genes, suggesting that these genes may be up- or downregulated in a concerted manner. Together, our studies depict a molecular trajectory with critical genes identified during chemical reprogramming of astrocytes into neurons.

### The Transcriptome Profile of Human Astrocytes

Our HAs were purchased from ScienCell (San Diego) and originated from fetal tissue. Therefore, one important question is whether there are neural progenitor cells contaminated in our astrocyte cultures that might account for neuronal conversion. To minimize neural progenitor contamination, we have passaged HAs for > 10 generations in the presence of 10% serum to fully differentiate any potential progenitor cells (Yin et al., 2019). In this study, we have compared our HA transcriptome profile to that reported for HAs (Zhang Y. et al., 2016) or neural stem cells (Modrek et al., 2017). Based on the gene expression profile and signaling pathway status, the HAs we used were much closer to other reported human fetal astrocytes than neural stem cells (NSCs). Specifically, our HA cultures were negative for neural TFs such as NEUROD1, NEUROG2, and TBR1, and also negative for non-astrocyte markers such as DCX, IBA1, NG2, or MBP, consistent with the nature of astrocytes with rare contamination of progenitor cells (Gao et al., 2017). In addition, we also found that cultured HAs displayed a certain level of reactivity. For example, several markers identified for the A1 type of reactive astrocytes, e.g., C3, GBP2, HSPB1, SERPING1, and TIMP1, were highly expressed in our astrocytes, together with active TGF-β and JAK/STAT signaling pathways. Our *in vivo* reprogramming work using TF NeuroD1 has found that reactive astrocytes under injury or disease conditions are more likely converted into neurons than the resting astrocytes (Guo et al., 2014). The fact that our cultured HAs bear certain reactive properties might explain why our chemical reprogramming approach can achieve quite a high conversion efficiency, around 70% (Yin et al., 2019).

### The Initial Signaling Cascade Involved in Chemical Reprogramming

Our transcriptome analyses found that the most DEGs among our chemical reprogramming process were occurring within 24 h of small molecule treatment, suggesting that the astrocytes respond in a profound way to the four small molecules (CHIR99021, DAPT, LDN193189, and SB431542). This is consistent with other studies using TFs or small molecules to convert astrocytes or fibroblast cells into neurons (Wapinski et al., 2013; Masserdotti et al., 2015), which all showed rapid changes in transcriptome profile within 24 h. During our chemical reprogramming process, one salient group of DEGs that were upregulated at D1 was related to hedgehog signaling, including ARHGAP36, PTCH1, HHIP, FGF19, ZNF521, and GLI1. All these changes contributed to the activation of the hedgehog pathway, which is well known to play a critical role during neuronal development and neural differentiation (Lai et al., 2003; Traiffort et al., 2010). Importantly, none of the four small molecules used in our work is a direct modulator on hedgehog, yet the synergistic effects of the four small molecules together result in a potent activation of the hedgehog pathway, suggesting that the interactions among GSK-3, Notch, BMP, and TGF-β will further activate much broader signaling cascades that will promote neuronal fate determination. Besides hedgehog signaling pathway, small molecules can have a direct effect on TFs. For example, GSK-3 inhibits NEUROG2 activity in cell culture and in the neocortex (Li S. et al., 2012), and we found here that GSK-3 inhibitor CHIR99021 can activate NEUROG2. Similarly, others in the bHLH family of neural TFs such as NEUROD1/2/6, NEUROG1, and HES5 were also upregulated during the chemical reprogramming process, suggesting that the combination of the four small molecules together can potently activate transcriptional machinery. This transcriptional activation by four small molecules is consistent with our previous findings using a nine-small-molecule cocktail for chemical reprogramming (Zhang et al., 2015) and another group using six small molecules for chemical reprogramming (Gao et al., 2017). Opposite to the upregulation of the hedgehog signaling pathway and neural TFs, the astroglial genes were rapidly downregulated. For instance, both FN1 and COL1A1 were highly expressed in astrocytes but drastically downregulated after the application of small molecules. Thus, the rapid activation of hedgehog signaling and bHLH TFs, together with the downregulation of glial genes, triggers the whole transcriptome change from an astrocytic profile toward a neuronal profile.

It is worth pointing out that the neural progenitor marker genes such as SOX2 and NES are also transiently upregulated at D1 together with many other neural TFs such as NEUROG1/2 and TBR1 upon small molecule treatment. However, these neural progenitor genes failed to further increase during the rest of the chemical reprogramming process, whereas neuron-related genes continued to be upregulated at D3–D14 and thus changing astrocytic transcriptomes directly intro neuronal transcriptomes without the neural progenitor stage. In support of this notion is the observation of a uniform decrease of cell cycle–related genes such as CCND1, CDKN1A, and CCNH at D1 upon drug application. Therefore, while small molecule treatment upregulated SOX2 and NES at D1, it also simultaneously inhibited cell proliferation, preventing astrocytes from turning into neural progenitors. Instead, the continuous increase in the expression level of bHLH factors together with many other neuronal genes will reprogram the astrocytes directly into neuronal cells.

### Gene Network Coordinating Chemical Reprogramming

One interesting finding that emerged from our gene network analyses based on highly expressed and highly up- or downregulated DEGs is the identification of a series of hub genes such as RGMA and GAP43 that may play a critical role in orchestrating the chemical reprogramming process. RGMA belongs to the family of repulsive guidance molecules, known for its role in axon guidance (Matsunaga, 2006). RGMA has also been linked to cancer cells (Li J. et al., 2012). Our gene network analyses revealed that RGMA may be connected with many neurogenic genes including GAP43, NEUROG2, JAG1, NNAT, PENK, NPTX2, MOXD1, and TBR1. More surprisingly, RGMA is also connected with glial genes such as TGFB1, IGFBP4, SERPINE1, and FN1. Since the expression level of RGMA in HAs is very low, such a link between RGMA and glial genes might represent a negative regulation of RGMA on glial genes. The growth cone–related gene GAP43 is also found here as a hub gene: it shows broad connections with glial genes in addition to the link with neurogenic genes. This is worth further study and may suggest new functional roles for these axon-related genes.

Besides RGMA and GAP43, NNAT is also widely connected with neurogenic genes as well as glial genes. NNAT is a lipid protein that regulates ion channels during neuronal differentiation (Lin et al., 2010). Our study found that NNAT is one of the most highly expressed genes among all DEGs during the chemical reprogramming process. The gene network analyses also revealed a close relationship between NNAT and RGMA. Moreover, NNAT is linked not only to neurogenic genes such as PENK, NEUROG2, GAP43, and TBR1, but also to glial genes such as IGFBP4 and TGFB1, suggesting that NNAT may have much broader functions than the previously reported regulation on ion channels.

In conclusion, our transcriptome analyses reveal a molecular cascade in response to small molecule application and depict a clear trajectory of gene profile shift during the AtN conversion process. The insight gained from these studies will help further refine a potential chemical therapy for neuroregeneration and neural repair.

## Experimental Procedures

### Conversion of Human Astrocytes Into Neurons

Human cortical astrocytes (HA1800 from ScienCell) were cultured as previously described (Guo et al., 2014; Zhang et al., 2015). Briefly, cells were cultured in HA medium containing DMEM/F12, 10% fetal bovine serum, B27 supplement, 3.5 mM glucose, 10 ng/ml fibroblast growth factor 2, 10 ng/ml epidermal growth factor, and penicillin–streptomycin. Astrocytes were cultured for 10–15 passages before seeded onto poly-D-lysine–treated coverslips in 24-well plates.

One day before chemical treatment, half of the HA medium was removed and substituted by N2 medium, which was composed of DMEM/F12, N2 supplement, and penicillin–streptomycin. After 24 h (day 0), the culture medium was totally changed into N2 medium with the four core drugs: CHIR99021 (1.5 μM), DAPT (5 μM), LDN193189 (0.25 μM), and SB431542 (5 μM). These chemicals were refreshed every 48 h on day 2 and day 4 together with N2 medium. To facilitate the survival and maturation of the converted neurons, on day 6, the culture medium was changed into differentiation medium including DMEM/F12, 0.5% fetal bovine serum, N2, B27, 5 mg/ml vitamin C, and penicillin–streptomycin. Neurotrophic factors such as 10 ng/ml BDNF, 10 ng/ml NT3, and 20 ng/ml IGF-1 were also added to the culture on day 6.

### RNA Extraction

HA samples were collected 1 day before drug treatment (HA sample), day 0 before drug treatment but with half N2 medium change, days 1, 3, 5 (days 1–5 during drug treatment), and day 14 after drug treatment (drug removal at day 6). Each time point contained three biological replicates. RNA extractions were performed using the Macherey-Nagel NucleoSpin^®^ RNA kit. Briefly, cells were lysed by 350 μl lysis buffer and 3.5 μl β-mercaptoethanol and then transferred to the NucleoSpin^®^ filter for binding and washing. The purified RNA was eluted with 40 μl RNase-free water. The concentration and purity of the samples were measured on NanoDrop^TM^ 2000 spectrophotometers (Thermo Fisher Scientific).

### RNA-Sequencing Analysis

RNA quality check, mRNA enrichment, library construction, and single-end 50 bp sequencing with HiSeq 3000 were performed at the UCLA Technology Center for Genomics and Bioinformatics. Quality checking of the raw data was done using FastQC (v. 0.11.3) using default settings. The filtered reads were aligned against human reference genome hg38 using HISAT2 (v. 2.0.1) (Kim et al., 2015) and summarized using featureCounts (v. 1.5.0) (Liao et al., 2014). Genes that had average expression levels greater than five counts were considered valid. Differential expression analysis was processed using DESeq2 (v. 1.16.1) (Love et al., 2014), and genes with more than threefold differences, baseMean > 80 and adjusted *p* < 0.01, were called DEGs. GO analysis was performed on Gene Ontology Consortium^1^, and enrichment plots were generated using GSEA (Subramanian et al., 2005). Gene expression changes in significant KEGG pathways were shown by Pathview (Luo and Brouwer, 2013). The correlation network was plotted using WGCNA (Langfelder and Horvath, 2008) and igraph (Csárdi and Nepusz, 2006).

## Data Availability

The datasets generated for this study can be found in GEO, GSE122701.

## Author Contributions

N-XM conducted the majority of the experiments, analyzed the data, and wrote the initial draft of manuscript. J-CY performed cell culture, immunostaining and quantification. GC supervised the entire project and revised the manuscript. All authors reviewed and approved the manuscript.

## Conflict of Interest Statement

GC was a co-founder of NeuExcell Therapeutics Inc. The remaining authors declare that the research was conducted in the absence of any commercial or financial relationships that could be construed as a potential conflict of interest.

## Footnotes

1 http://www.geneontology.org/
