# Supplementary material for: Transcriptome Analysis of Small Molecule–Mediated Astrocyte-to-Neuron Reprogramming
Source: Front Cell Dev Biol. 2019 May 31;7:82. doi: 10.3389/fcell.2019.00082 (PMC6558402; doi:10.3389/fcell.2019.00082)
Supplement: Supplementary file 1 [file Table_1.DOCX]

**Transcriptome Analysis of Small Molecule-Mediated**

**Astrocyte-to-Neuron Reprogramming**

Ning-Xin Ma, Jiu-Chao Yin, Gong Chen^†^

**Supplementary Figures and Legends**

**
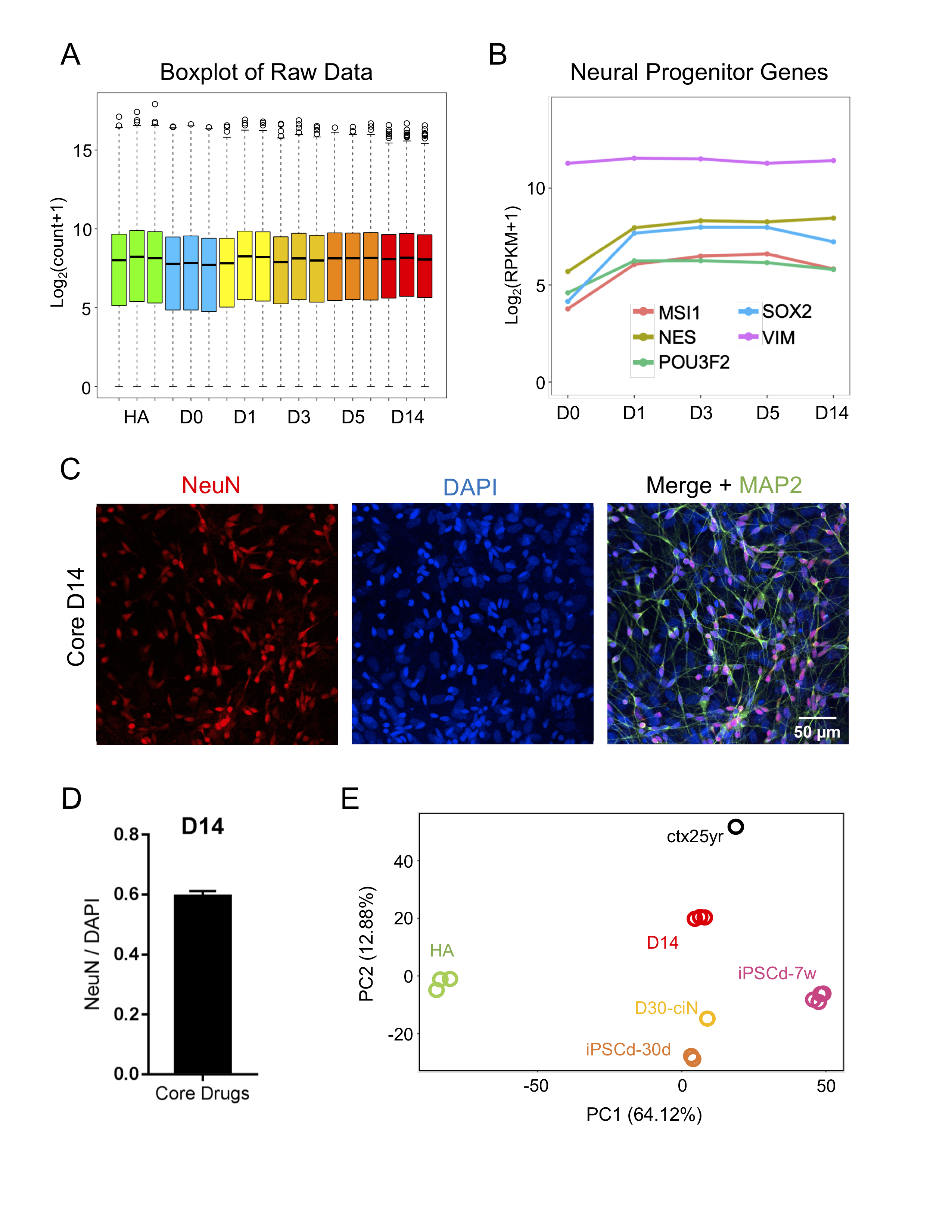
**

**Figure S1. Quality control and transcriptome comparison with available datasets.**

(A) Distribution of raw RNA-seq datasets among all samples. Boxplots represent log2 of the expression level for all 18 samples from 6 time points.

(B) A slight increase of neural progenitor cell markers was induced by core drugs. NES = Nestin. MSI1 = Musashi. POU3F2 = Brn2. VIM = Vimentin.

(C) Immunostaining of neuronal markers NeuN and MAP2 at D14.

(D) Quantification result of the NeuN/DAPI ratio (59.4% ± 1.8%).

(E) PCA analysis of our HA (green) and D14 (red) samples, together with previously reported datasets, including chemical induced 30-day neuron (yellow, GSE84826), iPSC-derived 30-day neuron (orange, GSE102352), iPSC-derived 7-week neuron (magenta, GSE88773), and 25-yr cortex tissue (black, GSE73721).


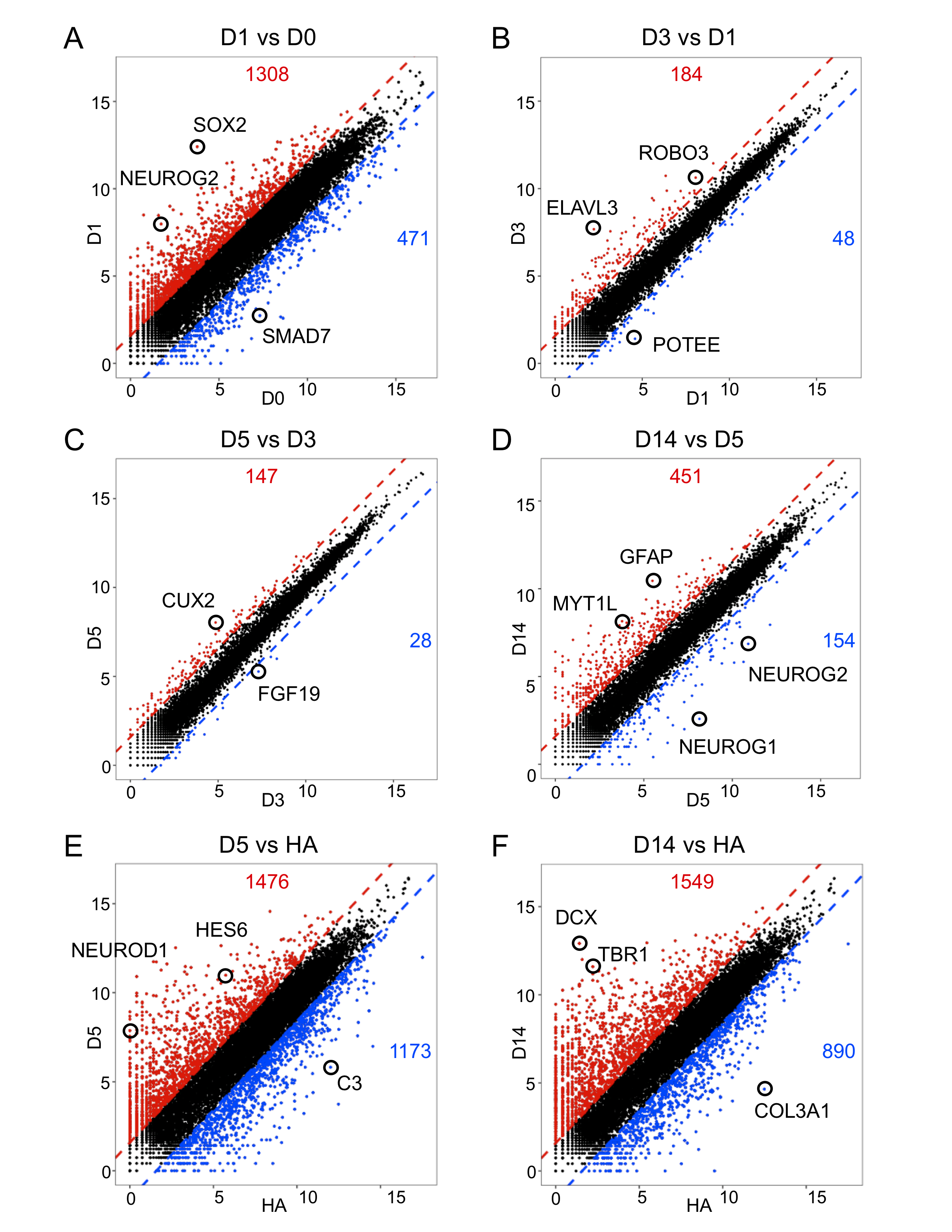


**Figure S2. Pair-wise gene expression comparisons between different samples.**

(A) Scatterplot represents the value of expression level (log2 read count) for each gene in D1 versus D0 samples. Genes with greater than 3-fold changes were labeled in red (higher in D1) or blue (higher in D0). The colored numbers correspond to the number of colored dots. Representative genes are circled and annotated.

(B-F) Scatterplots of pair-wise comparisons between D3 vs D1, D5 vs D3, D14 vs D5, D5 vs HA, and D14 vs HA.


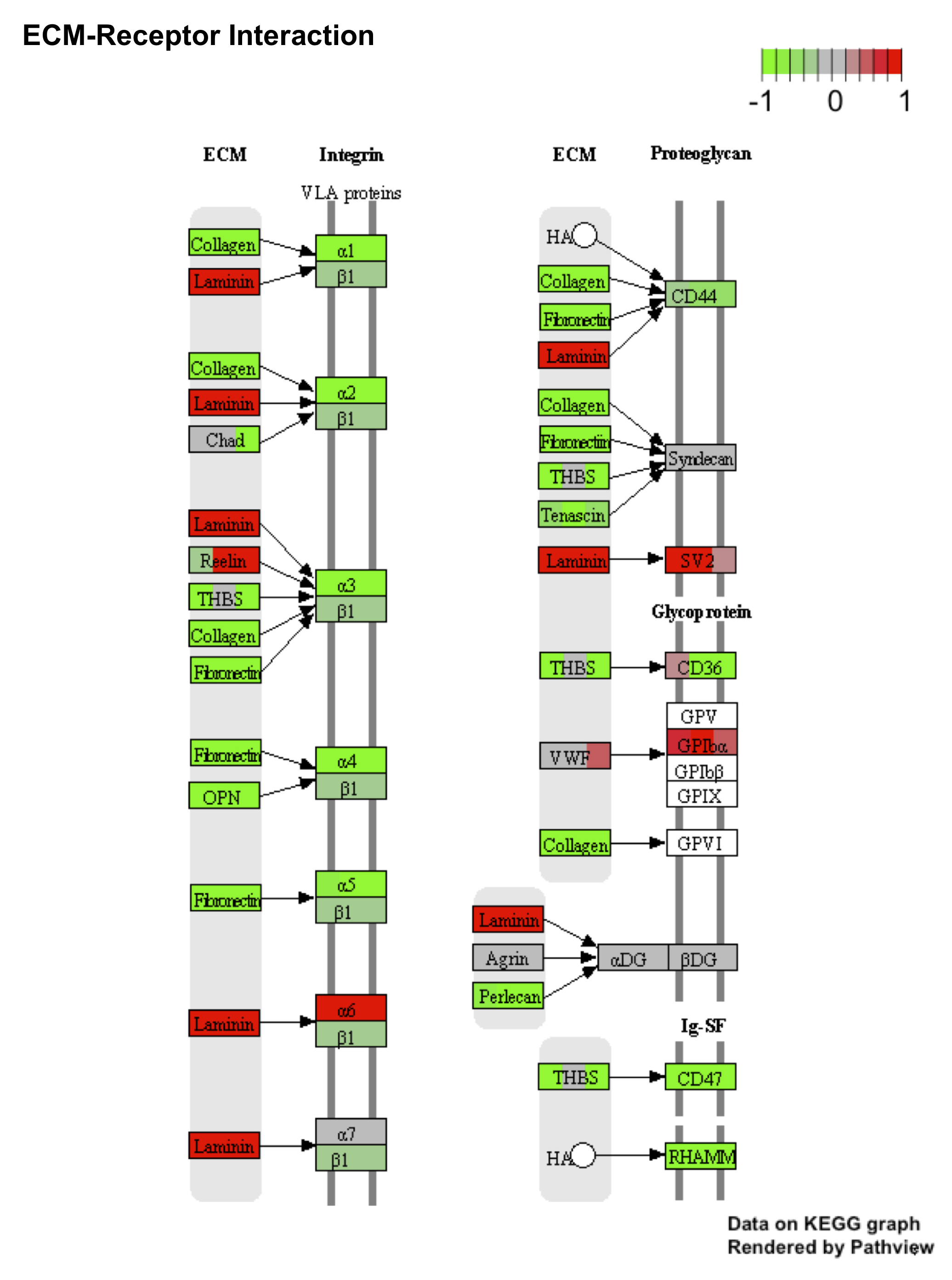


**Figure S3. Downregulation of extracellular matrix pathway during chemical reprogramming process.**

KEGG pathway analysis was used to compare DEGs between D1 and HA samples.

ECM pathway changes included the activation of laminin (red color means upregulation) and suppression of collagen and fibronectin (green color means downregulation).


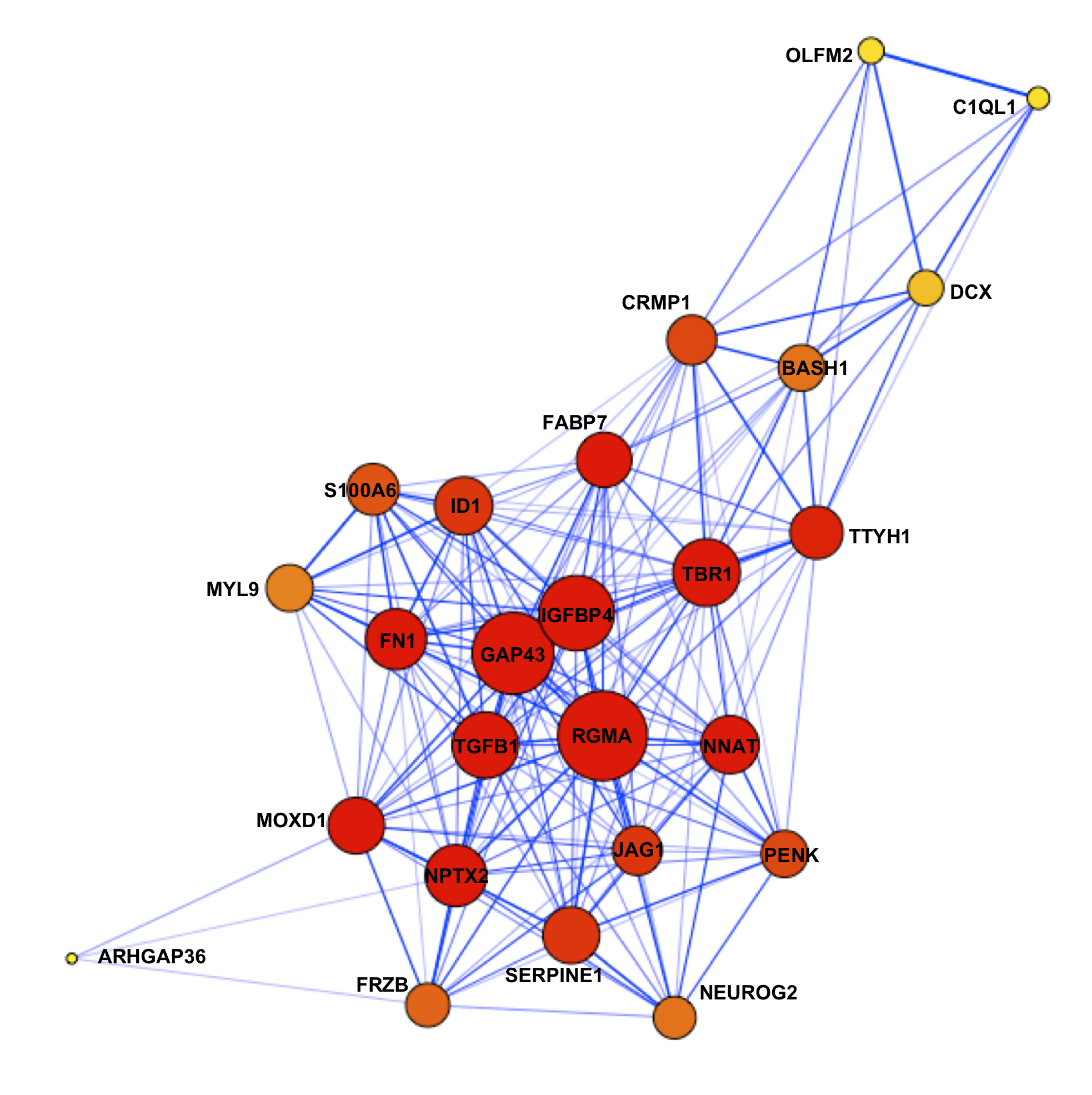


**Figure S4. Gene co-expression network based on top 25 DEGs.**

The correlation network was plotted by igraph.

Node color and line thickness represent different degree of connectivity.

Node size = weighted degree of connectivity.

This dataset is the same as Figure 7B but in different presentation format to show the weighted degree of connectivity for each gene.
